# Anti-Diabetic Atherosclerosis by Inhibiting High Glucose-Induced Vascular Smooth Muscle Cell Proliferation via Pin1/BRD4 Pathway

**DOI:** 10.1155/2020/4196482

**Published:** 2020-07-23

**Authors:** Yuansheng Wu, Meijin Zhang, Changsheng Xu, Dajun Chai, Feng Peng, Jinxiu Lin

**Affiliations:** ^1^The First Clinical Medical College, Fujian Medical University, Fuzhou, Fujian, China; ^2^Department of Cardiology, the First Affiliated Hospital, Fujian Medical University, Fuzhou, Fujian, China; ^3^Fujian Provincial Institute of Hypertension, the First Affiliated Hospital, Fujian Medical University, Fuzhou, Fujian, China

## Abstract

**Methods:**

Diabetic *Apoe*-/- mice induced by streptozotocin were treated with vehicle, the Pin1 inhibitor juglone, or the BRD4 inhibitor JQ1 for 3 weeks. VSMCs were pretreated with juglone, JQ1, or vehicle for 45 min, and then exposed to high glucose for 48 h. Hematoxylin–eosin staining was performed to assess atherosclerotic plaques of the thoracic aorta. Western blotting was used to detect expression levels of Pin1, BRD4, cyclin D1, and matrix metalloproteinase-9 (MMP-9) in the thoracic aorta and VSMCs. The 3-(4,5-dimethylthiazol-2-yl)-2,5-diphenyltetrazolium bromide (MTT) assay and transwell assay were used to measure proliferation and migration of VSMCs.

**Results:**

Juglone and JQ1 significantly improved atherosclerosis of diabetic *Apoe*-/- mice and reduced high glucose-induced VSMC proliferation and migration. Cyclin D1 and MMP-9 levels in the thoracic aorta were lower in diabetic *Apoe*-/- mice treated with juglone and JQ1 compared with vehicle-treated diabetic *Apoe*-/- mice. Additionally, BRD4 protein expression in high glucose-induced VSMCs was inhibited by juglone and JQ1. Upregulation of Pin1 expression by transduction of the Pin1 plasmid vector promoted BRD4 expression induced by high glucose, and stimulated proliferation and migration of VSMCs.

**Conclusions:**

Inhibition of Pin1/BRD4 pathway may improve diabetic atherosclerosis by inhibiting proliferation and migration of VSMCs.

## 1. Introduction

Atherosclerotic cardiovascular disease, including myocardial infarction and stroke, is the main cause of death in diabetes [1], but its mechanism is complicated. Studies have shown that abnormal proliferation and migration of vascular smooth muscle cells (VSMCs) from the middle layer of the artery to the endometrium is an important process for accelerating cardiovascular complications in diabetes [2]. Therefore, inhibition of abnormal proliferation and migration of VSMCs is important for preventing and treating diabetic atherosclerosis. However, the molecular mechanism involved in promoting proliferation and migration of VSMCs in the diabetic state is unclear.

Phosphorylation of serine or threonine prior to proline (pSer/Thr-Pro) is a common cellular signaling pathway during cell proliferation and differentiation [3]. Pin1 uniquely recognizes the pSer/Thr-Pro motif to catalyze its cis-trans isomerization. This affects the structure of many target proteins, regulating their phosphorylation level, catalytic activity, stability, subcellular localization, and mutual interaction with DNA, RNA, or other proteins [4–6]. Previous studies have shown that upregulation of Pin1 expression is associated with mitochondrial reactive oxygen species production and mitochondrial damage through interaction with the phosphorylated adaptor protein p66Shc and endothelial nitric oxide synthase. This then leads to endothelial dysfunction and vascular inflammation [7–9]. Additionally, the Pin1 inhibitor juglone is effective in inhibiting diabetic vascular lesions [10], which is a brown dye isolated from the shell and leaves of the walnut tree [11].

As a member of the bromodomain and extra-terminal structural family, bromine domain protein 4 (BRD4) remains bound to chromatin and is critical for regulating the cell cycle and proliferation [12]. BRD4 can recruit important transcription factors to the transcription initiation site, and enrich and control enhancer activity in the enhancer region. [13, 14] BRD4 also acts as an active kinase to phosphorylate RNA polymerase II, thereby directly or indirectly regulating transcription. Current research on BRD4 has focused on tumor and inflammation-related diseases [15, 16]. BRD4 also plays an important role in pathological cardiac hypertrophy, pulmonary hypertension, and proliferation of vascular endothelial cells and smooth muscle cells [17–19]. JQ1 is a highly selective small molecule inhibitor of BRD4. JQ1 blocks recruitment of BRD4 to chromatin by specifically interfering with binding of the BET bromodomain to acetylated lysine. Previous studies have shown that JQ1 significantly attenuates early atherosclerosis of high cholesterol-fed low-density lipoprotein (LDL) receptor knockout mice. Additionally, JQ1 inhibits expression of endothelial cell adhesion factor (E-selectin, vascular cell adhesion molecule-1) and cytokines (monocyte chemoattractant protein 1, interleukin-8), adhesion of monocytes to the endothelium, and rolling and migration of leukocytes [20]. These studies suggest that imbalance or dysfunction of BRD4 may be involved in progression of diabetic vasculopathy.

Pin1 binds directly to phosphorylated threonine (T) 204 of BRD4, preventing BRD4 ubiquitination and improving its stability. Pin1 also enhances the transcriptional activity of BRD4 by catalyzing the conformational change of proline 205 of BRD4 and enhancing the interaction between BRD4 and CDK9. In contrast, inhibition of Pin1 reduces the stability and transcriptional activity of BRD4, thereby inhibiting proliferation, migration, and invasion of tumor cells, and ultimately attenuating its carcinogenic ability [21]. Therefore, Pin1 may play an important role in abnormal proliferation and migration of smooth muscle cells in diabetic atherosclerosis by regulating BRD4.

In this study, we aimed to investigate whether juglone treatment inhibits BRD4 levels in the thoracic aorta and attenuates development of atherosclerosis in diabetic *Apoe*-/- mice. We also investigated whether inhibition of Pin1 improves high glucose-induced abnormal proliferation and migration of VSMCs by inhibiting BRD4 at the cellular level and its underlying mechanism.

## 2. Materials and Methods

### 2.1. Reagents and Kits

The following drugs were used: streptozotocin (STZ) (Sigma–Aldrich, St. Louis, MO); juglone (Pin1 inhibitor) (Sigma–Aldrich, St. Louis, MO); JQ1 (BRD4 inhibitor) (Sigma–Aldrich, St. Louis, MO). The following antibodies were used: mouse matrix metalloproteinase 9 (MMP-9) monoclonal antibody (1: 1000, Servicebio, GB12132-1), mouse *β*-actin monoclonal antibody (1: 2000, Santa Cruz, sc-47778), mouse cyclin D1 monoclonal antibody (1: 1000, Santa Cruz, sc-8396), rabbit Pin1 polyclonal antibody (1: 2000, Millipore, 07-091), rabbit BRD4 monoclonal antibody (1: 1000, Abcam, ab128874), horseradish peroxidase-conjugated goat anti-rabbit IgG (secondary antibody) (1: 8000), and horseradish peroxidase-conjugated goat anti-mouse IgG (secondary antibody) (1: 8000). Polyvinylidenefluoride (PVDF) membranes were purchased from Millipore. M199 medium and fetal bovine serum (FBS) were from Gbico, and the Liposome Transfection Kit was from Invitrogen.

### 2.2. Animals

The experiments were approved by the Experimental Animal Ethics Committee of Fujian Medical University and they were carried out in accordance with the institutional guidelines. Fifty male mice weighing 20 ± 2 g were provided by Beijing Huarong Kang Biotechnology Co., Ltd. (Beijing, China), including 10 C57BL/6 mice and 40 *Apoe*-/- mice (License number: SCXK) 2014-0004). To establish a diabetic animal model, 30 *Apoe*-/- mice were intraperitoneally injected by single-dose STZ (100 mg/kg in 0.025 M citrate buffer, pH 4.5). After 72 h, mice with fasting blood glucose levels >16.7 mM were considered diabetic. The mice were then randomly assigned to the DM group (vehicle, *n* =10), the juglone group (juglone 1 mg/kg/d intraperitoneally every 3 days, *n* =10), and the JQ1 group (JQ1 50 mg/kg/d intraperitoneally every other day, *n* =10). The remaining 10 male *Apoe*-/- mice were selected as the *Apoe*-/- group (*n* =10). The C57BL/6 group (C57BL/6 mice), *Apoe*-/- group, and DM group were treated with intraperitoneal injection of the same amount of normal saline. All of the mice were housed in cages under the same temperature (20-22°C), fed freely, and maintained in a 12-h light/dark cycle. After 3 weeks, blood samples were obtained by retroperitoneal puncture under barbital-induced anesthesia after 12 h of fasting, and aliquots of serum were stored at 4°C for analysis.

### 2.3. Cell Culture

VSMCs were cultured by the tissue adherence method, and only 3–6 passages were used for experiments. More than 98% of the cells were positive for smooth muscle-specific *α*-actin staining and showed typical VSMC hill and valley morphology. Cells grown to 80%–95% confluence were allowed to stand for 24 h by starvation (0.3% FBS). Subsequently, all cells were divided into blank control group (normal glucose 5.5 mM), hypertonic group (normal glucose + mannitol, 19.5 mM), high glucose (25 mM) group, juglone (high glucose + juglone, 10^−5^ M) group, JQ1 (high glucose + JQ1, 10^−5^ M) group.

### 2.4. Hematoxylin–Eosin Staining of the Thoracic Aorta

After administration of sodium pentobarbital (50 mg/kg, intraperitoneal injection), the mice were euthanized, and the thoracic aorta was isolated and fixed with 10% formaldehyde buffer for 48 h. After dehydration, transparency, and paraffin embedding, the tissue was cut into thin slices of 4 *μ*m in thickness and then subjected to conventional hematoxylin–eosin (HE) staining. Plaques were observed under a microscope and photographed, and the plaque area was measured by the computer-aided Image Pro Plus 6.0 software (*Media Cybernetics*, *Inc., Rockville, MD, USA*).

### 2.5. Western Blotting

The same amount of protein from each sample (mouse thoracic aorta homogenate or VSMC lysate) was *electrophoresed* using 12% sodium dodecyl sulfate-polyacrylamide gel electrophoresis and transferred onto PVDF membranes. The membranes were then incubated with blocking buffer at room temperature (5% dry milk, 0.1% Tween 20, in TBS) for 2 h and primary antibody overnight at 4°C. After incubation with a suitable secondary antibody, the immunoreactive bands were visualized and analyzed using a FluorChem E exposure imaging analysis system (Cell Biosciences, USA).

### 2.6. Real-Time Reverse Transcription-Polymerase Chain Reaction

Total RNA from VSMCs was extracted with Trizol reagent (Invitrogen) and reverse transcribed using a reverse transcription-polymerase chain reaction (RT-PCR) kit (Takara, Dalian, China). PCR primers (Shanghai Boshang Company) were as follows: *β*-actin: 5′-AGAGGGAAATCGTGCGTGAC-3′ (forward) and 5′-GAAGGAAGGCTGGAAGAGAG-3′ (reverse); and Pin1: 5′-CGCAAATGGGCGGTAGGCGTG-3′ (forward) and 5′-CCTCTACAAATGTGGTATGGC-3′ (reverse). For RT-PCR, SYBR from Premix Ex TaqTM (Takara, Dalian, China) was used, and real-time RT-PCR conditions were based on the product specifications. The final PCR products were subjected to gradient temperature-dependent dissociation to verify that only one product was amplified. Reactions without RT sample or template were used as negative controls. Relative quantitative assessment of target gene levels was performed by comparing cycle thresholds and reactions were performed in triplicate.

### 2.7. Plasmid Design and Synthesis

The plasmid vector clone was purchased from Weizhen Biotechnology Co., Ltd. The logarithmic growth phase of VSMCs (approximately 5 × 10^5^ per well) was synchronized in the culture medium of 0.3% FBS for 24 h. After adding M199 medium containing the plasmid and liposome, the cells were further incubated for 24 h, and a new medium containing 0.3% FBS was replaced for 24 h. The intervention experiment was then performed.

### 2.8. MTT Assay

Cell proliferation assays were performed by using the 3-(4,5-dimethylthiazol-2-yl)-2,5-diphenyltetrazolium bromide (MTT) method. Briefly, growth-stable VSMCs were incubated with or without intervention for 24 h, and the cultures were discarded and incubated with 5 mg/mL MTT for 4 h at 37°C. Finally, the medium was removed, and the crystals were dissolved in 150 *μ*L of dimethyl sulfoxide, followed by shaking at room temperature for 10 min. The absorbance was read at a wavelength of 490 nm with the iMark plate reader (Bio-Rad, USA).

### 2.9. Cell Migration Assay

The modified Boyden microporous membrane double groove method was used for determining cell migration [22]. The migration membrane with a pore size of 4.5 *μ*m was boiled in 0.1% gelatin for 1 h. After drying the membrane, the rough surface of the migration membrane was numbered with a pencil. A total of 0.3 mL induction culture solution was added to the lower chamber and the migration membrane was placed on the culture solution of the lower chamber. A volume of 0.4–0.5 mL of VSMC suspension was seeded in the upper chamber (total number of cells: 1 × 10^5^). After 24 h of incubation at 37°C in a 5% CO_2_ incubator, the cell migration membrane was fixed with methanol and cells on the lower side were stained with toluidine blue. The number of blue-stained cells was randomly calculated in 5 small circled areas of the migration membrane (10× objective lens, *n* =6).

### 2.10. Statistical Analysis

Results are presented as mean ± SD. The results were analyzed by one-way factorial ANOVA followed by post hoc comparisons. All statistics were performed by SPSS version 16 (SPSS, Chicago, IL, USA). A *P* value <0.05 was considered statistically significant.

## 3. Results

### 3.1. Effect of Juglone and JQ1 Treatment on Lipid Metabolism in Diabetic *Apoe*-/- Mice

**Figure 1:**
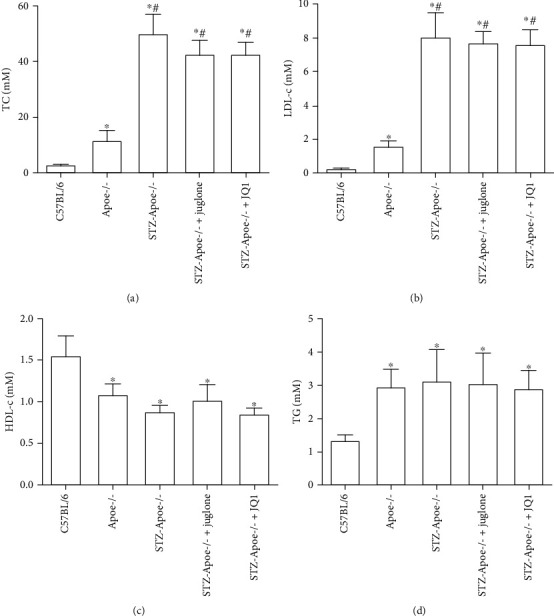
Effect of juglone and JQ1 treatment on lipid metabolism in diabetic *Apoe*-/- mice. Values are mean ± SEM, with *n* =10 mice per group. Abbreviations: TC, total cholesterol; TG, triglycerides; HDL-c, high-density lipoprotein cholesterol; LDL-c, low-density lipoprotein cholesterol. ^∗^*P* < 0.05 vs. C57BL/6; ^#^*P* <0.05 vs. *Apoe*-/-.

Levels of LDL-cholesterol (LDL-c), total cholesterol (TC), and triglycerides (TG) in *Apoe*-/- mice were significantly higher, but high-density lipoprotein cholesterol (HDL-c) levels were lower, compared with C57BL/6 mice (all *P* <0.05). Levels of LDL-c and TC in diabetic *Apoe*-/- mice were significantly higher than those in *Apoe*-/- mice (both *P* <0.05), with no significant difference in HDL-c and TG levels. Compared with diabetic *Apoe*-/- mice, levels of blood lipid were no significant difference after treatment with juglone or JQ1 (all *P* >0.05) (Figure 1).

### 3.2. Effect of Juglone and JQ1 Treatment on Thoracic Aortic Plaques in Diabetic *Apoe*-/- Mice

**Figure 2:**
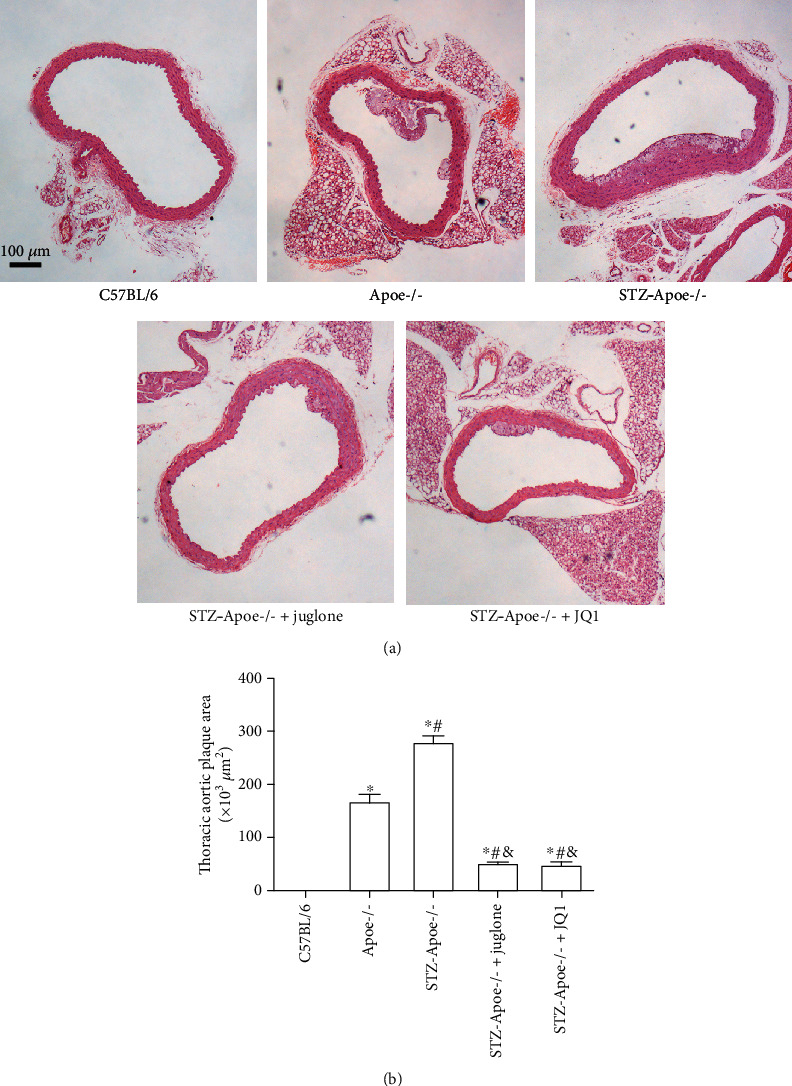
Effect of juglone and JQ1 treatment on thoracic aortic plaques in diabetic *Apoe*-/- mice. (a). HE staining of the mouse thoracic aorta (scale bars =100 *μ*m). In the C57BL/6 group, no plaques were found in the intima of the thoracic aorta, the intima was smooth and flat, and the thickness of the middle membrane was uniform. The *Apoe*-/- group showed lipid deposition, a rough intima, and an irregular media. In the DM group, large plaques were found, the inner membrane was rough and discontinuous, and cells at the bottom of plaques were prominently proliferated and irregular. There was only lipid deposition in the intima of the thoracic aorta in the juglone group. The intima was smoother in the juglone group than in the DM group, with a small amount of hyperplasia at the lipid base. Only small plaques were found in the thoracic aorta lumen in the JQ1 group. Additionally, cells at the top of plaques proliferated to form fibrous caps, and there was only a small amount of cell proliferation at the bottom of plaques. (b). Analysis of the plaque area in HE-stained thoracic aorta of mice. Values are mean ± SEM, with *n* =10 mice per group. ^∗^*P* < 0.05 vs. C57BL/6; ^#^*P* <0.05 vs. *Apoe*-/-;^&^*P* <0.05 vs. STZ-*Apoe*-/-.

Compared with C57BL/6 mice, the plaque area in all other groups was significantly higher (all *P* <0.05). The plaque area in diabetic *Apoe*-/- mice was significantly higher than that in *Apoe*-/- mice (*P* <0.05). Juglone and JQ1 treatment reduced the thoracic aortic plaque area in STZ-induced diabetic *Apoe*-/- mice (both *P* <0.05). There was no significant difference in aortic plaque area between the juglone and JQ1 groups (Figure 2).

### 3.3. Effect of Juglone and JQ1 Treatment on Pin1, BRD4, Cyclin D1, and MMP-9 Expression in the Thoracic Aorta in Diabetic *Apoe*-/- Mice

**Figure 3:**
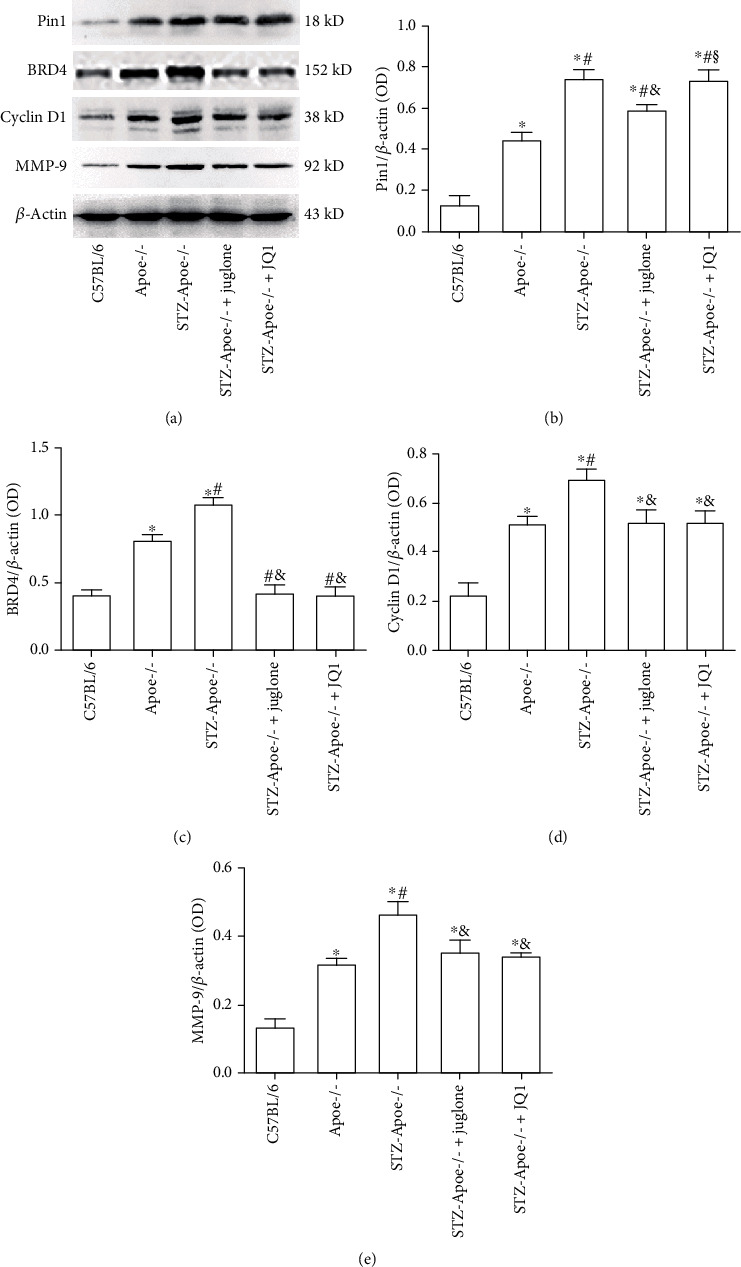
Effect of juglone and JQ1 treatment on Pin1, BRD4, cyclin D1, and MMP-9 protein levels in the thoracic aorta of diabetic *Apoe*-/- mice. (a) Immunoblot analysis was performed on homogenates from thoracic aorta tissue of C57BL/6 mice, *Apoe*-/- mice, and diabetic *Apoe*-/- mice treated with vehicle, juglone, or JQ1. Pin1, BRD4, cyclin D1, and MMP-9 protein levels were determined by western blotting. (b-e) Relative ratios of Pin1, BRD4, cyclin D1, and MMP-9 over *β*-actin were determined by densitometric analysis. Values are mean ± SEM (*n* =10 mice per group). ^∗^*P* < 0.05 vs. C57BL/6; ^#^*P* <0.05 vs. *Apoe*-/-;^&^*P* <0.05 vs. STZ-*Apoe*-/-; ^§^*P* <0.05 vs. STZ-*Apoe*-/- + juglone.

Pin1, BRD4, cyclin D1, and MMP-9 protein expression levels were higher in the thoracic aorta of the *Apoe*-/- mice compared with the C57BL/6 mice, and even higher in the diabetic *Apoe*-/- mice (all *P* <0.05). Both juglone and BRD4 treatment alleviated BRD4, cyclin D1, and MMP-9 expression in the thoracic aorta of diabetic *Apoe*-/- mice. Compared with the DM group, juglone treatment significantly reduced Pin1 expression in the thoracic aorta of diabetic *Apoe*-/- mice (*P* <0.05), but JQ1 treatment had no effect (*P* >0.05) (Figure 3).

### 3.4. Effects of Juglone and JQ1 Treatment on Protein Expression of Pin1, BRD4, Cyclin D1, and MMP-9 in VSMCs Induced by High Glucose

Western blotting showed that high glucose (11.1-33 mM) promoted Pin1 protein expression of VSMCs in a dose-dependent manner (Supplemental Figure 1(a)). Compared with control VSMCs, Pin1 protein expression in VSMCs induced by high glucose at 24 h was significantly higher (*P* <0.05), and it was even higher at 48 h (*P* <0.05), but there was no difference between 48 and 72 h (Supplemental Figure 1(b)). Juglone inhibited high glucose-induced Pin1 protein expression in VSMCs in a concentration-dependent manner. With pretreatment of VSMCs with juglone (10^−7^–10^−5^ M) for 45 min and incubation with high glucose for 48 h, Pin1 protein expression in the juglone 10^−5^ M group was reduced the most *(P* <0.05, Supplemental Figure 2(a)). JQ1 (10^−7^–10^−5^ M) inhibited high glucose-induced BRD4 protein expression in VSMCs in a concentration-dependent manner. BRD4 protein expression in the JQ1 10^−5^ M group was reduced the most (*P* <0.05, Supplemental Figure 2(b)).

**Figure 4:**
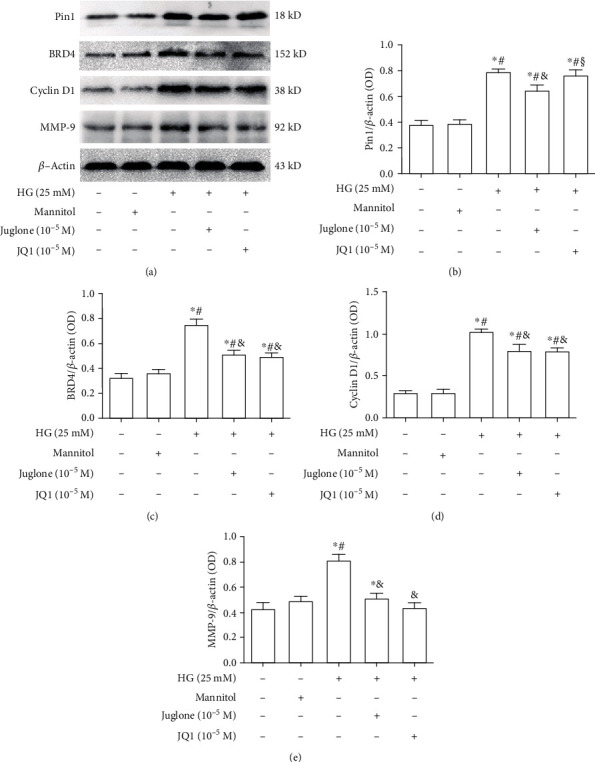
Effect of juglone and JQ1 treatment on Pin1, BRD4, cyclin D1, and MMP-9 protein expression in high glucose-induced VSMCs. (a) Effects of juglone and JQ1 treatment on Pin1, BRD4, cyclin D1, and MMP-9 protein expression in high glucose-cultured VSMCs. (b-e) Relative ratios of Pin1, BRD4, cyclin D1, and MMP-9 over *β*-actin were determined by densitometric analysis. Values are mean ± SEM (*n* =6, 6 experiments per group). HG, high glucose (25 mM). ^∗^*P* < 0.05 vs. blank control group (normal glucose, 5.5 mM); ^#^*P* <0.05 vs. hypertonic group (normal glucose 5.5 mM + mannitol 19.5 mM); ^&^*P* <0.05 vs. HG (25 mM); ^§^*P* <0.05 vs. HG + juglone 10^−5^ M.

VSMCs were pretreated with juglone (10^−5^ M) or JQ1 (10^−5^ M) for 45 min, and then high glucose (25 mM) was added for 48 h. Western blotting showed that Pin1, BRD4, cyclin D1, and MMP-9 protein expression levels of high glucose-treated VSMCs were significantly higher compared with those in control VSMCs (all *P* <0.05). Increased BRD4, cyclin D1, and MMP-9 protein expression levels were reduced by juglone and JQ1 (all *P* <0.05). Only juglone reduced Pin1 protein expression, with no significant change after JQ1 treatment. There were no significant differences in cyclin D1 and MMP-9 protein expression levels between the juglone and JQ1 groups (Figure 4).

### 3.5. Effects of Juglone and JQ1 Treatment on Proliferation and Migration of VSMCs Induced by High Glucose

**Figure 5:**
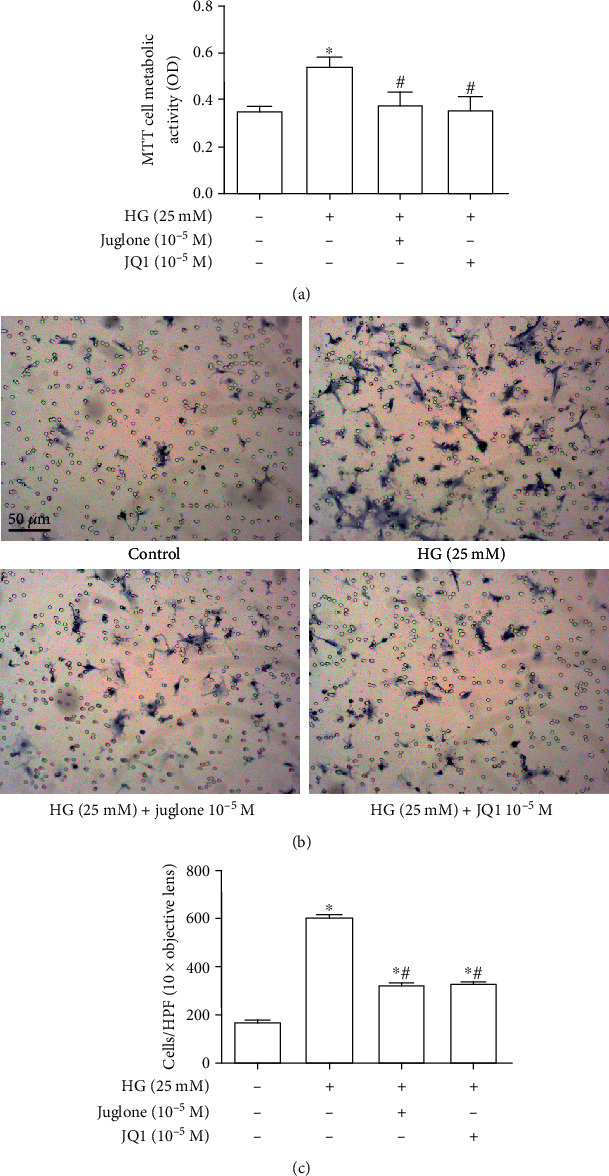
Effect of juglone and JQ1 treatment on proliferation and migration of VSMCs induced by high glucose. (a) Effects of juglone and JQ1 treatment on proliferation of VSMCs induced by high glucose. VSMCs were inoculated in 96-well plates. When there was 60%–70% confluence, M199 medium containing 0.3% FBS was replaced and cultured for 24 h. Pretreatment with juglone (10^−5^ M) or JQ1 (10^−5^ M) was performed for 45 min, and then high glucose (25 mM) was added with incubation for 24 h. After this time, 10 *μ*L MTT was added and incubated at 37°C for 4–5 h. Optical density was detected at 490 nm. (b, c) Effects of juglone and JQ1 treatment on migration of VSMCs induced by high glucose. Vehicle or high glucose (25 mM) was added to cells with or without the Pin1 inhibitor juglone (10^−5^ M) or the BRD4 inhibitor JQ1 (10^−5^ M) to the lower chamber. After 24 h of incubation at 37°C in a 5% CO2 incubator, the cell migration membrane was fixed with methanol and stained with toluidine blue. Cell migration was observed under a microscope (scale bars =50 *μ*m). Values are mean ± SEM (*n* =6, 6 experiments per group). ^∗^*P* < 0.05 vs. Control; ^#^*P* <0.05 vs. HG (25 mM).

The MTT assay showed that the metabolic activity of VSMCs induced by high glucose (25 mM) was significantly higher compared with that in control VSMCs (*P* <0.05). Both juglone (10^−5^ M) and JQ1 (10^−5^ M) abrogated the increased metabolic activity of VSMCs under the high glucose condition (both *P* <0.05, Figure 5(a)). The cell migration test showed that the number of migrated VSMCs after high glucose treatment was significantly higher compared with that in control VSMCs (*P* <0.05). Juglone and JQ1 significantly reduced migration of VSMCs induced by high glucose (both *P* <0.05, Figures 5(b) and 5(c)).

### 3.6. Pin1 Overexpression Mediates the Effects on Protein Expression Levels of BRD4, Cyclin D1, and MMP-9, and Proliferation and Migration of VSMCs Induced by High Glucose

**Figure 6:**
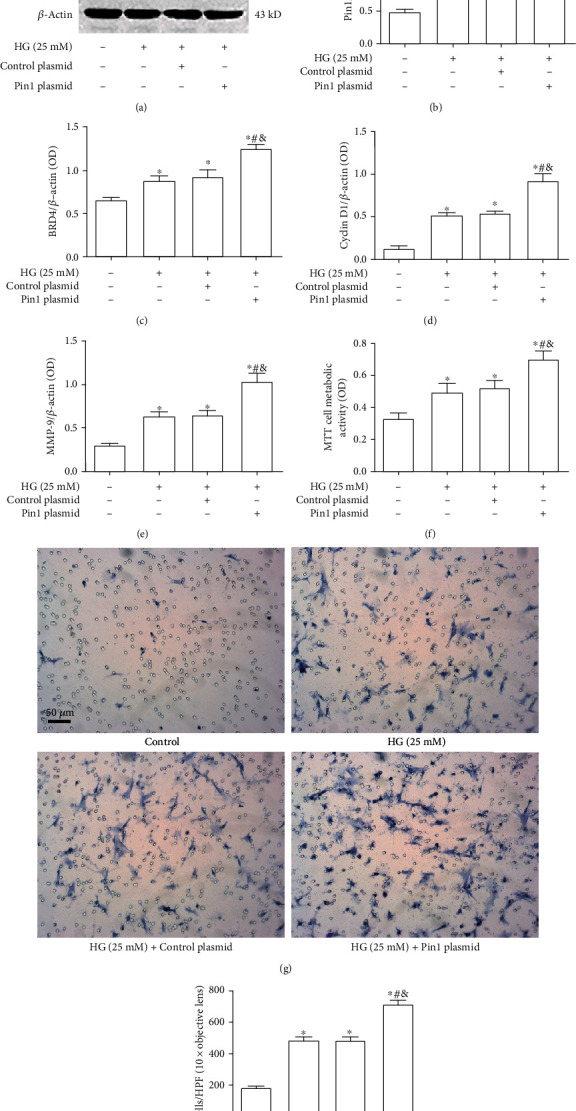
Effects on Pin1, BRD4, cyclin D1, and MMP-9 protein expression levels, and proliferation and migration of VSMCs induced by high glucose when Pin1 is overexpressed. (a) Effects on Pin1, BRD4, cyclin D1, and MMP-9 protein expression levels in VSMCs induced by high glucose when Pin1 is overexpressed. Pin1 plasmid vector was transduced to VSMCs by the liposome method and treated with or without high glucose (25 mM) for 48 h. Cell protein was extracted for western blotting. (b-e) Relative ratios of Pin1, BRD4, cyclin D1, and MMP-9 over *β*-actin were determined by densitometric analysis. (f) Effects on proliferation of VSMCs induced by high glucose when Pin1 is overexpressed. The proliferation ability of cells was determined by the MTT assay (the experimental process was as described above). (g, h) Effects on migration of VSMCs induced by high glucose when Pin1 is overexpressed. The migration ability of cells was determined by the transwell assay (the experimental process was as described above) (scale bars =50 *μ*m). Values are mean ± SEM (*n* = 6, 6 experiments per group). ^∗^*P* < 0.05 vs. Control; ^#^*P* < 0.05 vs. HG (25 mM); ^&^*P* < 0.05 vs. HG (25 mM) + juglone 10^−5^ M.

Protein and mRNA expression levels of Pin1 in the empty plasmid group were not significantly different compared with control VSMCs, which were significantly increased in VSMCs of Pin1 plasmid transduction (*P* < 0.05, Supplemental Figure 3). After VSMCs were transduced with the Pin1 plasmid vector, the effect on the increase in Pin1, BRD4, cyclin D1, and MMP-9 protein expression in VSMCs induced by high glucose was further elevated, accompanied by increasement of cellular metabolic activity and migration ability (all *P* < 0.05, Figure 6).

## 4. Discussion

Cell proliferation, chronic inflammation, and oxidative stress play an important role in diabetic atherosclerosis. Many studies have shown that abnormal proliferation and migration of VSMCs from the middle layer of the arteries to the endometrium are associated with the process of diabetic atherosclerosis [2]. Furthermore, Pin1 regulates proliferation of VSMCs, apoptosis, and progression of the cell cycle through the *β*-catenin/cyclin D1/CDK4 pathway and mitochondrial pathway [23]. BRD4 is regulated by Pin1 [21]. Therefore, we speculate that Pin1 and BRD4 are important regulatory proteins of diabetes that promote macrovascular disease, but the underlying mechanism is still unclear.

In the current study, we found that Pin1 and BRD4 protein expression in the thoracic aorta of diabetic *Apoe*-/- mice and in VSMCs induced by high glucose were significantly increased. Juglone inhibited Pin1 and BRD4 expression, accompanied by decreased cyclin D1 and MMP-9 protein levels. These findings suggest that regulation of Pin1 and BRD4 expression affects proliferation and migration of VSMCs in the development of diabetic atherosclerosis. Additionally, we found that the Pin1 inhibitor juglone inhibited BRD4 expression, and the BRD4 inhibitor JQ1 had no significant effect on Pin1 expression, which further confirmed that BRD4 was regulated by Pin1. Our findings on the effects of Pin1 and BRD4 in diabetic atherosclerosis are consistent with previous studies [10, 20], but this study is the first to examine the mechanism of the Pin1/BRD4 pathway at different cellular levels.

Our study suggested that juglone did not cause lipid metabolism disorders in diabetic *Apoe*-/- mice, which is consistent with a study by Liu et al. [24]. Our study also showed that the blood lipid levels of diabetic Apoe-/- mice are no significant difference after treatment with the JQ1. Like our study, Brown et al. used JQ1 to interfere with LDL receptor knockout mice and found that this improved atherosclerosis without affecting blood lipid levels [20]. These findings suggest that juglone and JQ1 have additional effects on atherosclerosis, which may improve atherosclerosis by regulating proliferation and migration of VSMCs.

Many studies have shown that cyclin D1 activation is closely related to proliferation of VSMCs and remodeling of the endothelium. Upregulation of Pin1 expression in cells enhances cyclin D1 gene expression by activating c-Jun/activator protein-1, *β*-catenin/T-cell factor, and nuclear factor-*kappaB* transcription factors. Pin1 also directly binds to the pThr286-Pro motif on cyclin D1 to stabilize nuclear cyclin D1 [25]. Additionally, mouse Pin1 knockout produces a phenotype similar to cyclin D1 knockout [26]. Studies have also shown that BRD4 regulates cyclin D1 expression [27, 28]. Our study showed that juglone and JQ1 inhibited cyclin D1 protein expression induced by high glucose. This finding suggests that Pin1/BRD4 affects smooth muscle cell proliferation by regulating cyclin D1. Changes in phenotype in smooth muscle cells are important pathophysiological mechanisms of atherosclerosis [29]. Smooth muscle cells with a synthetic phenotype can secrete many extracellular matrix proteins and increase migration ability [30]. When VSMCs switch from the contractile phenotype to the proliferative migration phenotype, MMPs are secreted and inflammatory cytokines are produced to promote vascular remodeling [31]. Studies have shown that Pin1 and RBD4 interact with NF-*κ*B and cause increased expression of MMP genes [32, 33]. Our study showed that juglone and JQ1 inhibited increased MMP-9 protein expression induced by high glucose, which is consistent with studies by Liang and Duan et al. [34, 35]. This finding suggests that Pin1/BRD4 affects smooth muscle cell migration by regulating MMP-9.

In conclusion, our study shows that the Pin1/BRD4 pathway may affect proliferation and migration of VSMCs by regulating expression of cyclin D1 (regulatory protein of proliferation) and MMP-9 (regulatory protein of migration). This could ultimately affect the occurrence of diabetic atherosclerosis. Antagonizing the Pin1/BRD4 pathway may be a feasible method for preventing and treating macrovascular disease in patients with diabetes. These findings will provide a theoretical and experimental basis for the development of new drugs for patients with diabetes.

## Data Availability

The data used to support the findings of this study are available from the corresponding author upon request.

## Supplementary Materials

Supplementary Materials Supplemental Figure 1: High glucose increases Pin1 protein expression of VSMCs in a dose- and time-dependent manner. Supplemental Figure 2: Juglone and JQ1 reduce upregulation of Pin1 and BRD4 protein expression induced by high glucose in a dose-dependent manner. Supplemental Figure 3: Effects of Pin1 plasmid vector transduction on Pin1 mRNA and protein expression levels in VSMCs.

## References

[1] Low Wang C. C., Hess C. N., Hiatt W. R., Goldfine A. B. (2016). Clinical update: cardiovascular disease in diabetes mellitus: atherosclerotic cardiovascular disease and heart failure in type 2 diabetes mellitus-mechanisms, management, and clinical considerations. *Circulation*.

[2] Beckman J. A., Creager M. A., Libby P. (2002). Diabetes and atherosclerosis: epidemiology, pathophysiology, and management. *JAMA*.

[3] Poli A., Mongiorgi S., Cocco L., Follo M. Y. (2014). Protein kinase C involvement in cell cycle modulation. *Biochemical Society Transactions*.

[4] Nicholson L. K., Lu K. P. (2007). Prolyl _cis_ - _trans_ Isomerization as a Molecular Timer in Crk Signaling. *Molecular Cell*.

[5] Lu K. P., Zhou X. Z. (2007). The prolyl isomerase PIN1: a pivotal new twist in phosphorylation signalling and disease. *Nature Reviews Molecular Cell Biology*.

[6] Liou Y. C., Zhou X. Z., Lu K. P. (2011). Prolyl isomerase Pin1 as a molecular switch to determine the fate of phosphoproteins. *Trends in Biochemical Sciences*.

[7] Lv L., Zhang J., Zhang L. (2013). Essential role of Pin1 via STAT3 signalling and mitochondria-dependent pathways in restenosis in type 2 diabetes. *Journal of Cellular and Molecular Medicine*.

[8] Paneni F., Costantino S., Castello L. (2015). Targeting prolyl-isomerase Pin1 prevents mitochondrial oxidative stress and vascular dysfunction: insights in patients with diabetes. *European Heart Journal*.

[9] Zhang M., Lin L., Xu C., Chai D., Peng F., Lin J. (2018). VDR agonist prevents diabetic endothelial dysfunction through inhibition of prolyl Isomerase-1-mediated mitochondrial oxidative stress and inflammation. *Oxidative Medicine and Cellular Longevity*.

[10] Costantino S., Paneni F., Lüscher T. F., Cosentino F. (2016). Pin1 inhibitor Juglone prevents diabetic vascular dysfunction. *International Journal of Cardiology*.

[11] Hennig L., Christner C., Kipping M. (1998). Selective inactivation of parvulin-like Peptidyl-Prolylcis/transIsomerases by Juglone†. *Biochemistry*.

[12] You J., Li Q., Wu C., Kim J., Ottinger M., Howley P. M. (2009). Regulation of aurora B expression by the bromodomain protein Brd 4. *Molecular and Cellular Biology*.

[13] Zhang W., Prakash C., Sum C. (2012). Bromodomain-containing protein 4 (BRD4) regulates RNA polymerase II serine 2 phosphorylation in human CD4+ T cells. *Journal of Biological Chemistry*.

[14] Kanno T., Kanno Y., LeRoy G. (2014). BRD4 assists elongation of both coding and enhancer RNAs by interacting with acetylated histones. *Nature Structural & Molecular Biology*.

[15] Dey A., Yang W., Gegonne A. (2019). BRD4 directs hematopoietic stem cell development and modulates macrophage inflammatory responses. *EMBO Journal*.

[16] Mu J., Sun P., Ma Z., Sun P. (2019). BRD4 promotes tumor progression and NF-*κ*B/CCL2-dependent tumor-associated macrophage recruitment in GIST. *Cell Death & Disease*.

[17] Spiltoir J. I., Stratton M. S., Cavasin M. A. (2013). BET acetyl-lysine binding proteins control pathological cardiac hypertrophy. *Journal of Molecular and Cellular Cardiology*.

[18] Mumby S., Gambaryan N., Meng C. (2017). Bromodomain and extra-terminal protein mimic JQ1 decreases inflammation in human vascular endothelial cells: implications for pulmonary arterial hypertension. *Respirology*.

[19] Dutzmann J., Haertle M., Daniel J. M. (2020). BET bromodomain containing epigenetic reader proteins regulate vascular smooth muscle cell proliferation and neointima formation. *Cardiovascular Research*.

[20] Brown J. D., Lin C. Y., Duan Q. (2014). NF-*κ*B directs dynamic super enhancer formation in inflammation and atherogenesis. *Molecular Cell*.

[21] Hu X., Dong S. H., Chen J. (2017). Prolyl isomerase PIN1 regulates the stability, transcriptional activity and oncogenic potential of BRD4. *Oncogene*.

[22] Vipra M. R., Chiplonkar J. M. (2002). Vital stain to study cell invasion in modified Boyden chamber assay. *BioTechniques*.

[23] Lv L., Zhou Z., Huang X. (2010). Inhibition of peptidyl-prolyl cis/trans isomerase Pin1 induces cell cycle arrest and apoptosis in vascular smooth muscle cells. *Apoptosis*.

[24] Liu M., Yu P., Jiang H. (2017). The essential role of Pin1 via NF-*κ*B signaling in vascular inflammation and atherosclerosis in ApoE-/- mice. *International Journal of Molecular Sciences*.

[25] Wulf G., Garg P., Liou Y. C., Iglehart D., Lu K. P. (2004). Modeling breast cancer in vivo and ex vivo reveals an essential role of Pin1 in tumorigenesis. *The EMBO Journal*.

[26] Liou Y. C., Ryo A., Huang H. K. (2002). Loss of Pin1 function in the mouse causes phenotypes resembling cyclin D1-null phenotypes. *Proceedings of the National Academy of Sciences of the United States of America*.

[27] Xiang T., Bai J. Y., She C., Yu D. J., Zhou X. Z., Zhao T. L. (2018). Bromodomain protein BRD4 promotes cell proliferation in skin squamous cell carcinoma. *Cellular Signalling*.

[28] Fiskus W., Sharma S., Qi J. (2014). Highly active combination of BRD4 antagonist and histone deacetylase inhibitor against human acute myelogenous leukemia cells. *Molecular Cancer Therapeutics*.

[29] Shankman L. S., Gomez D., Cherepanova O. A. (2015). KLF4-dependent phenotypic modulation of smooth muscle cells has a key role in atherosclerotic plaque pathogenesis. *Nature Medicine*.

[30] Hultgårdh-Nilsson A., Lövdahl C., Blomgren K., Kallin B., Thyberg J. (1997). Expression of phenotype- and proliferation-related genes in rat aortic smooth muscle cells in primary culture. *Cardiovascular Research*.

[31] Orr A. W., Hastings N. E., Blackman B. R., Wamhoff B. R. (2010). Complex regulation and function of the inflammatory smooth muscle cell phenotype in atherosclerosis. *Journal of Vascular Research*.

[32] Qin L. Y., Li M. N., Ren W. J. (2010). Silencing Pin1 suppresses the expression and bioactivity of MMP-9 through NF-*κ*B in colorectal carcinoma SW480 cells. *Clinical Oncology and Cancer Research*.

[33] Wu S. Y., Nin D. S., Lee A. Y., Simanski S., Kodadek T., Chiang C. M. (2016). BRD4 phosphorylation regulates HPV E2-mediated viral transcription, origin replication, and cellular *MMP-9* expression. *Cell Reports*.

[34] Duan Q., Mao X., Liao C. (2016). Inhibition of BET bromodomain attenuates angiotensin II induced abdominal aortic aneurysm in ApoE^−/−^ mice. *International Journal of Cardiology*.

[35] Liang E., Cheng W., Yang R. X., Bai W. W., Liu X., Zhao Y. X. (2018). Peptidyl-prolyl isomerase Pin1 deficiency attenuates angiotensin II-induced abdominal aortic aneurysm formation in ApoE^−/−^ mice. *Journal of Molecular and Cellular Cardiology*.

