# Supplementary material for: Anti-Diabetic Atherosclerosis by Inhibiting High Glucose-Induced Vascular Smooth Muscle Cell Proliferation via Pin1/BRD4 Pathway
Source: Oxid Med Cell Longev. 2020 Jul 23;2020:4196482. doi: 10.1155/2020/4196482 (PMC7396119; doi:10.1155/2020/4196482)
Supplement: Supplementary Materials — Supplemental Figure 1: High glucose increases Pin1 protein expression of VSMCs in a dose- and time-dependent manner. Supplemental Figure 2: Juglone and JQ1 reduce upregulation of Pin1 and BRD4 protein expression induced by high glucose in a dose-dependent manner. Supplemental Figure 3: Effects of Pin1 plasmid vector transduction on Pin1 mRNA and protein expression levels in VSMCs. [file 4196482.f1.docx]

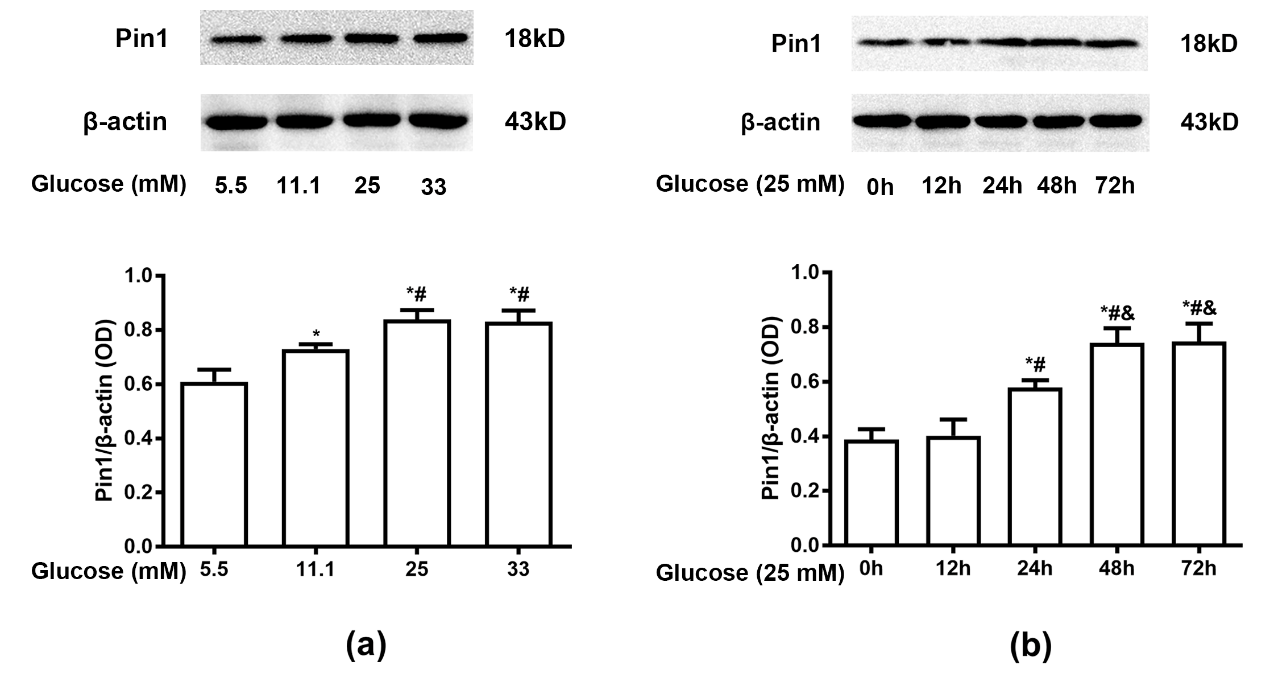


Supplemental figure 1: High glucose increases Pin1 protein expression of VSMCs in a dose- and time-dependent manner. (a) High glucose increased Pin1 protein expression of VSMCs in a dose-dependent manner. VSMCs were inoculated into 6-well culture plates. When 70%–80% confluence was achieved, medium containing 0.3% FBS was replaced and different concentrations of high glucose (11.1-33 mM) were added to the culture for 24 h. VSMCs were extracted for western blotting to measure Pin1 protein levels. The relative ratio of Pin1 over β-actin was determined by densitometric analysis. Values are mean ± SEM (*n* = 6). **P* < 0.05 vs. Glucose 5.5 mM; ^#^*P* < 0.05 vs. Glucose 11.1 mM. (b) High glucose increased Pin1 protein expression of VSMCs in a time-dependent manner. VSMCs were treated with high glucose (25 mM) for 12, 24, 48, and 72 h. Pin1 protein expression was determined by western blotting. The relative ratio of Pin1 over β-actin was determined by densitometric analysis. Values are mean ± SEM (*n* = 6, 6 experiments per group). **P* < 0.05 vs. 0 h; ^#^*P* < 0.05 vs. 12 h; ^&^*P* < 0.05 vs. 24 h.


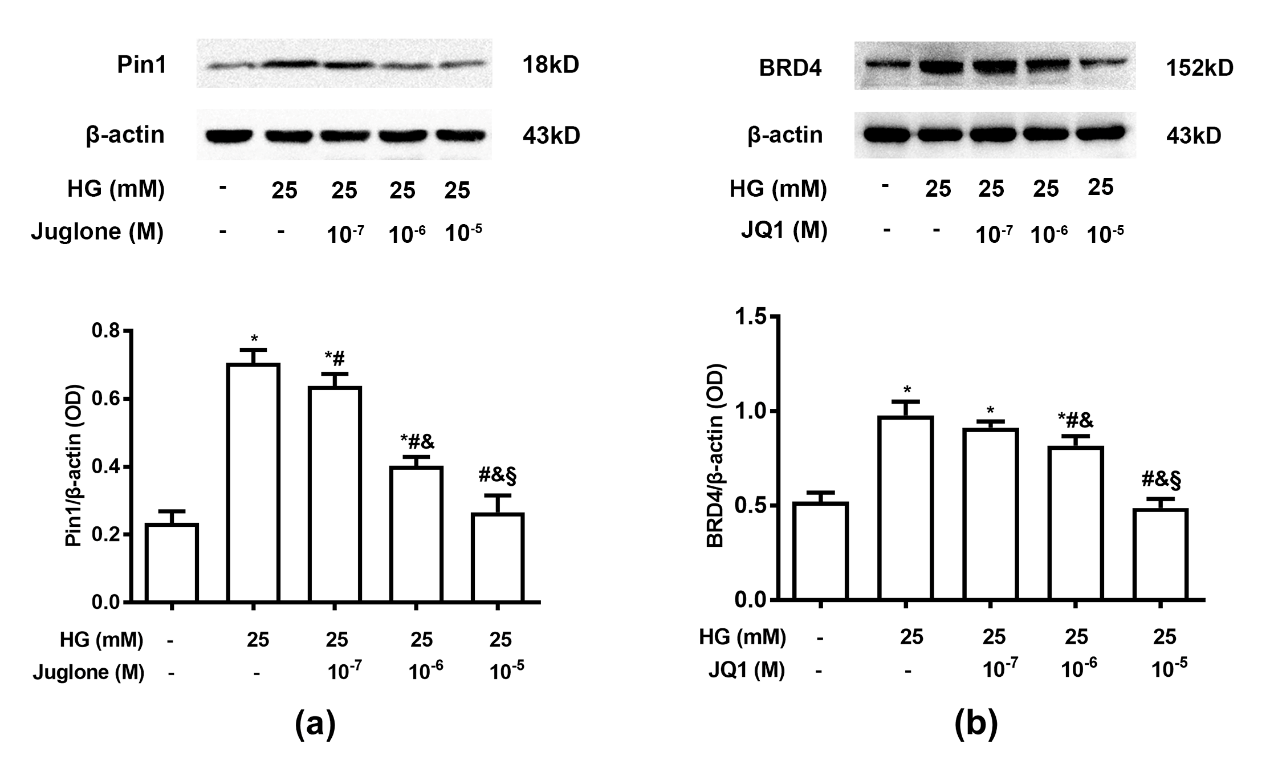


Supplemental figure 2: Juglone and JQ1 reduce upregulation of Pin1 and BRD4 protein expression induced by high glucose in a dose-dependent manner. (a) Juglone inhibited high glucose-induced upregulation of Pin1 protein expression in a dose-dependent manner. Pretreatment of VSMCs with different concentrations of juglone (10^-7^–10^-5^ M) was performed for 45 min. Incubation with high glucose (25 mM) for 48 h was then performed and VSMCs were extracted for western blotting. Pin1 protein expression levels of VSMC are expressed as the ratio of Pin1 over β-actin. Values are mean ± SEM (*n* = 6). **P* < 0.05 vs. Normal glucose 5.5 mM + juglone 0 M; ^#^*P* < 0.05 vs. HG 25 mM + juglone 0 M; ^&^*P* < 0.05 vs. HG 25 mM + juglone 10^−7^ M; ^§^*P* < 0.05 vs. HG 25 mM + juglone 10^−6^ M. (b) JQ1 inhibited high glucose-induced upregulation of BRD4 protein expression in a dose-dependent manner. VSMCs were pretreated with different concentrations of JQ1 (10^−7^–10^−5^ M) for 45 min and incubated with high glucose (25 mM) for 48 h. VSMCs were extracted for western blotting. BRD4 protein expression levels of VSMCs are expressed as the ratio of BRD4 over β-actin. Values are mean ± SEM (*n* = 6, 6 experiments per group). **P* < 0.05 vs Normal glucose 5.5 mM + JQ1 0 M; ^#^*P* < 0.05 vs. HG 25 mM + JQ1 0 M; ^&^*P* < 0.05 vs. HG 25 mM + JQ1 10^−7^ M; ^§^*P* < 0.05 vs. HG 25 mM + JQ1 10^−6^ M.


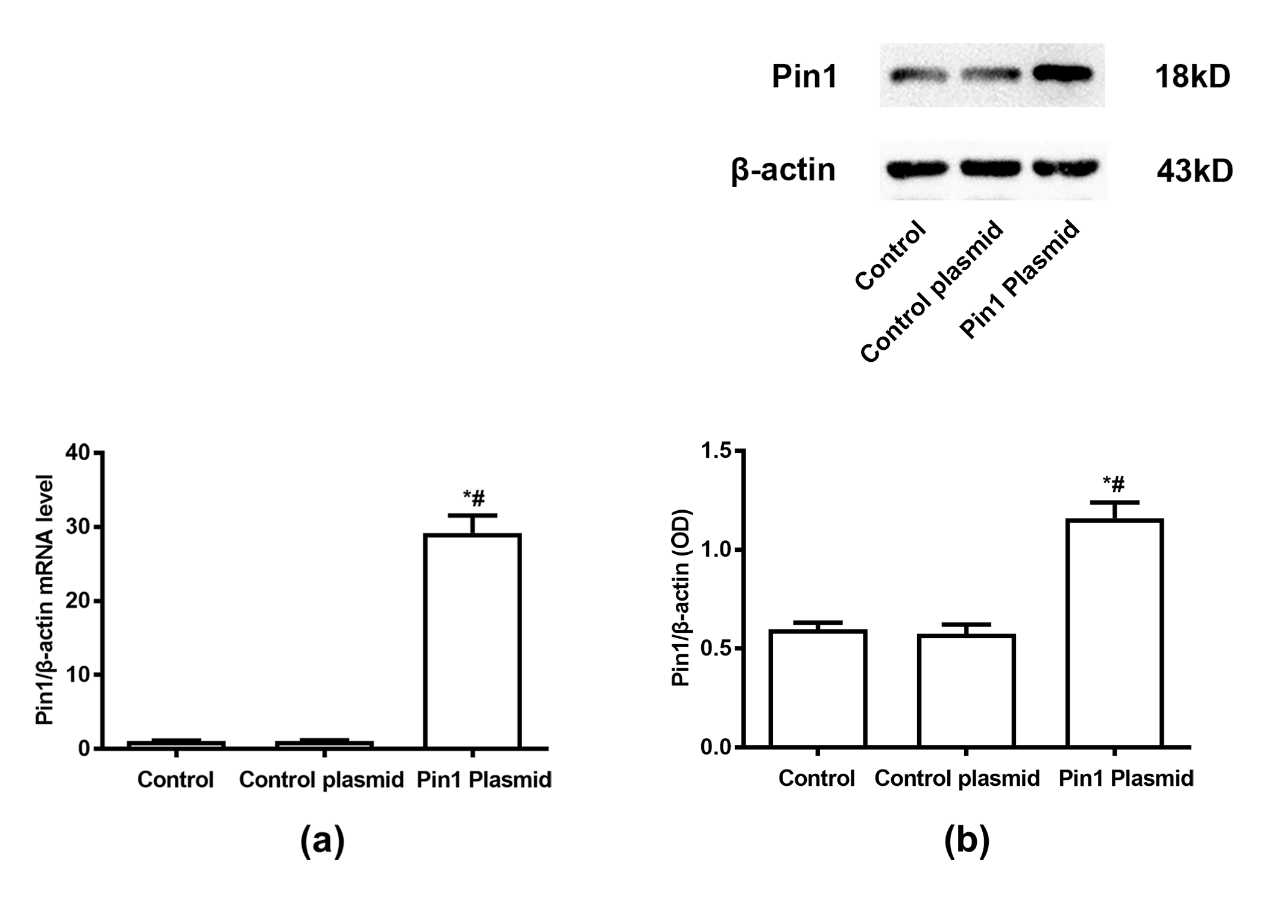


Supplemental figure 3: Effects of Pin1 plasmid vector transduction on Pin1 mRNA and protein expression levels in VSMCs. (a) Effects of Pin1 plasmid vector transduction on Pin1 mRNA expression levels in VSMCs. After the Pin1 plasmid vector was transduced into VSMCs by liposome for 48 h, total RNA of VSMCs was extracted and Pin1 mRNA expression levels were measured by real-time RT-PCR. (b) Effects of Pin1 plasmid vector transduction on Pin1 protein expression levels in VSMCs. VSMC total protein was extracted and Pin1 protein expression levels were measured by immunoblotting analysis. Values are mean ± SEM (n = 6, 6 experiments per grou p). **P* < 0.05 vs. control; ^#^*P* < 0.05 vs. control plasmid.
